# Olfactory Measures as Predictors of Conversion to Mild Cognitive Impairment and Alzheimer’s Disease

**DOI:** 10.3390/brainsci11111391

**Published:** 2021-10-23

**Authors:** Paul Loyd Wheeler, Claire Murphy

**Affiliations:** 1 Department of Psychology, San Diego State University, San Diego, CA 92182, USA; paul.loyd.wheeler@gmail.com; 2 Department of Psychiatry, San Diego School of Medicine, University of California, La Jolla, CA 92093, USA

**Keywords:** Alzheimer’s disease, biomarker, MCI, apolipoprotein E, odor threshold, odor identification, odor familiarity, odor memory

## Abstract

Background: Early biomarkers of prodromal Alzheimer’s disease (AD) are critical both to initiate interventions and to choose participants for clinical trials. Odor threshold, odor identification and odor familiarity are impaired in AD. Methods: We investigated the relative abilities of standard screening (MMSE) and olfactory measures to predict transitions from cognitively normal (CN) to mild cognitive impairment (MCI), from CN to AD, and MCI to AD. The archival sample of 497, from the UCSD ADRC, included participants who were CN, MCI, AD and converters to MCI or AD. Apoe ε4 status, a genetic risk factor, was available for 256 participants, 132 were ε4 carriers. A receiver operating characteristic curve (ROC) curve plots the trade-off between sensitivity and specificity. Area under the ROC curve (AUC) was used to determine diagnostic accuracy. Results: Different measures were better predictors at specific stages of disease risk; e.g., odor familiarity, odor identification and the combination showed higher predictive value for converting from MCI to AD in ε4 carriers than the MMSE. Combining odor familiarity and odor identification produced an AUC of 1.0 in ε4 carriers, MMSE alone was 0.58. Conclusions: Olfactory biomarkers show real promise as non-invasive indicators of prodromal AD. The results support the value of combining olfactory measures in assessment of risk for conversion to MCI and to AD.

## 1. Introduction

### 1.1. Alzheimer’s Disease

Alzheimer’s Disease (AD) is a neurodegenerative disease characterized by a significant decline in memory functions, personality changes, deterioration of language functions, motor dysfunction and death (DSM V Criteria Alzheimer’s Disease) [1]. It is estimated to affect more than 6 million Americans and 50 million people worldwide [2,3]. The number of people diagnosed with AD in the US alone is projected to increase to 13.8 million by 2060 [2]. Early detection will be critical as disease modifying drugs and health care treatments become available so that interventions can be initiated before profound neurological damage can occur.

### 1.2. Mild Cognitive Impairment (MCI)

In the progression from cognitively normal to AD, patients often pass through a transition stage of mild cognitive impairment (MCI) [4,5]. Though they show cognitive impairment, MCI patients are not demented, and activities of daily living remain largely preserved [4]. Dementia requires significant impairment and decline from a previous level of functioning that interferes with independence in activities of daily living in at least one of the following cognitive domains: learning and memory, language, complex attention, perceptual-motor function, social cognition. In one study, approximately 35% of patients with MCI transitioned to dementia within one year [6]. Other studies have indicated conversion rates of 15% to 24% within two years, or approximately one third over three years. However, transition rates have varied across studies [7,8]. Typically, MCI is assessed with neuropsychological testing such as the Mini-Mental Status Examination (MMSE) [9,10,11]. Advancements in the ability to predict progression from cognitively normal to MCI and MCI to dementia will be essential for clinical trial design and for patients to make treatment decisions [12,13].

### 1.3. Alzheimer’s Pathology

The characteristic pathological changes in AD include the amyloid-β (A-β) plaques and neurofibrillary tangles (NFT) first described by Dr. Alois Alzheimer in the brain of a woman who died with dementia. Tangles are aggregates of hyperphosphorylated tau that progressively accumulate in areas important for memory and cognition. Post mortem studies indicate that specific brain areas are affected early in AD, and suggest that the integrity of these areas can serve as biomarkers of AD. Braak and Braak identified six stages in the development of amyloid plaques and NFTs. NFTs develop very early in the locus coeruleus, transentorhinal and entorhinal areas, and then in the hippocampus and the limbic system. [14,15,16,17,18]. Importantly, the areas targeted early in AD tau pathology are also areas that are very important for processing olfactory information [19,20,21,22], and poorer odor identification has been associated with tau pathology [23].

Neurofibrillary tangles and neuritic plaques appear very early in the olfactory bulb and anterior olfactory nucleus [24,25]. Afferent neurons carry signals from the olfactory bulb into target brain regions including the entorhinal cortex, piriform cortex and amygdala [21,22,24]. Neuropathology in the entorhinal cortex would be expected to disconnect incoming olfactory information from the hippocampus and disrupt olfactory processing [1]. Neuroimaging indicates that the primary olfactory cortex, entorhinal cortex, and hippocampus degenerate at similar rates in Alzheimer’s and MCI patients [26]. Furthermore, decreased activation within the left primary odor cortex has been observed in both MCI and AD groups compared to healthy controls [26,27]. Thus, olfactory tasks that challenge these brain areas in MCI and AD patients may reflect AD pathology and serve as biomarkers of disease.

### 1.4. Apolipoprotein (ApoE) ε_4_

The apolipoprotein (ApoE) ε_4_ allele is associated with an increased risk for developing sporadic Alzheimer’s disease. The apolipoprotein gene has three alleles: ε2, ε3, and ε4. The presence of at least one ε4 allele increases the risk for AD [28]. Two copies of the ApoE ε4 allele (ε4/4 homozygotes) produce a higher risk for developing AD and more rapid cognitive decline [28]. For this reason, clinical drug trials are often enriched with ε4 carriers. Notably, ApoE ε_4_ carriers show impairments in olfactory function, with homozygotes showing the greatest impairment [29,30,31,32].

### 1.5. Olfaction in Alzheimer’s Disease

Although olfaction shows impairment with normal aging [33], the magnitude of the impairment in Alzheimer’s patients is profoundly greater than that of typically aging adults. Alzheimer’s patients show significantly greater deficits in odor thresholds, recognition memory, remote odor memory, discrimination, identification, olfactory event-related potentials and functional MRI during olfactory tasks than age matched controls [34,35,36,37,38,39]. Relative to non-carriers, Apoe ε4+ carriers have poorer odor identification, poorer episodic odor recognition memory, longer P3 latencies for olfactory stimuli, differential fMRI activation, and altered functional connectivity when processing odor memory information [1,32,40,41]. The imaging data suggest that Apoe ε4+ carriers expend greater cognitive effort for increased neural activation which is consistent with a compensatory hypothesis [42].

### 1.6. Odor Threshold

Odor threshold is a measure of olfactory sensory functioning. Odor thresholds are compromised in AD [34,43,44], and threshold levels are associated with the degree of dementia in AD patients [45]. Threshold decline often occurs shortly before the diagnosis of AD [29]. Although AD patients are often unaware of a deficit in smell sensitivity, odor thresholds in AD patients are, on average, approximately nine times the concentration required for age-matched controls [36].

### 1.7. Odor Identification and Odor Familiarity

Odor identification places demands on odor detection, verbal semantic memory and participant knowledge. It is well-established that AD patients exhibit significant impairments in odor identification [32,34,37,39,46,47,48]. Odor identification deficits have also been found in MCI [43,49], in those at risk for AD because of the ApoE e4 allele [31], and in first-degree relatives of AD patients [50,51].

Apoe ε4 carriers experience a more rapid decline in odor identification than odor threshold or dementia rating scale scores [52]. In a longitudinal study, over a ten-year period, Apoe ε4+ homozygotes experienced a decline in odor identification that was twice as fast as Apoe ε4+ heterozygote and non-carriers [30].

Patients with mild cognitive impairment who have both poor odor identification and lack of awareness of odor impairment show an increased risk for AD at follow up [7]. In one study by [53], difficulty identifying familiar odors was implicated in the subsequent development of mild cognitive impairment (MCI), a precursor to dementia in AD, and was robustly correlated with level of AD pathology upon post mortem evaluation. Risk of MCI was associated with odor identification test scores, and a person who made four errors was about 50% more likely to develop MCI than a person who made only one error [53]. Longitudinal population-based studies and research center investigations have shown that poor odor identification scores at baseline are associated with cognitive decline [54,55,56,57].

Two states of awareness are thought to be involved in recognition memory retrieval: recollection, or remembering an item along with context, and familiarity, where an item, in this case an odor, is recognized as familiar but not necessarily identified and no other contextual information is required to be remembered [58,59,60]. Familiarity is supported by the structures in the mesial temporal lobe that are very early affected in AD [58]. Because the neuropathology of AD occurs first in the mesial temporal areas, familiarity may reflect very early disease, and thus has potential as a biomarker for AD.

Nicolli-Waller et al. [35] demonstrated significant decreases in odor familiarity in Alzheimer’s patients. Patients with MCI also report less familiarity with odors [53].

### 1.8. Hypotheses

Olfaction shows real promise as a biomarker of AD, yet there are few studies investigating the sensitivity and specificity of olfactory measures for predicting transitions in the disease process. Because the neuropathology in AD progressively affects structures involved in olfactory processing, we hypothesized that olfactory tasks (odor threshold, odor identification, odor familiarity) would be differentially affected at distinct stages of disease progression: cognitively normal, but at risk for MCI and AD. Further, we hypothesized that combining more than one task might be expected to facilitate the detection of additional individuals at risk for conversion, because different olfactory tasks rely on different olfactory networks. Finally, we hypothesized that ε4+ carriers would show clearer predictions from the olfactory tasks.

## 2. Materials and Methods

### 2.1. Participants

**Figure 1:**
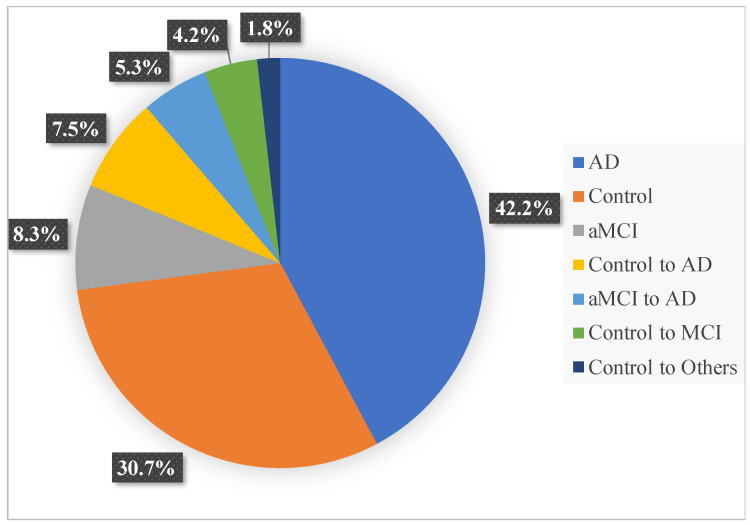
Percentages of the sample in each diagnostic category.

An archival sample of 497 participants, with odor threshold, identification and remote memory data, from the Alzheimer’s Disease Research Center longitudinal study at the University of California San Diego (UCSD ADRC) was used in the analysis. Patients had been recruited as they joined the longitudinal study at the UCSD ADRC. A consensus diagnosis was established by two different neurologists. Alzheimer’s disease diagnosis was based on Diagnostic and Statistical Manual of Mental Disorders, 5th Edition and NIA-Alzheimer’s Association (AA) diagnostic criteria, MCI was based on NIA-AA diagnostic criteria after extensive neuropsychological and clinical in person assessments. Patients with Parkinson’s disease were excluded. Patients were approached for olfactory assessment at their annual visit. The mean number of assessments was 2, but varied across the sample. The sample consisted of 49.1% women and 50.9% men. Of the participants, 42.2% had probable Alzheimer’s disease, 30.7% were cognitively normal controls, 8.3% were aMCI non-converters, 7.5% converted from cognitively normal control to AD, 5.3% converted from aMCI to AD, 4.2% converted from cognitively normal control to aMCI, and 1.8% converted from cognitively normal control to other (See Figure 1 for a graphic presentation of the sample at the point of the analysis). Informed consent was obtained from all participants. Two hundred fifty-six participants had had genomic testing; 51.6% of those participants were identified as Apoe ε4 carriers. 

### 2.2. Genotyping

Genomic DNA was obtained from blood samples. A polymerase chain reaction with oligonucleotide primers F4 and F6, followed by digestion of the HhA1 restriction enzyme, and polyacrylamide chain gel electrophoresis were used for genotyping [61,62]. Participants were then categorized as allele positive (having at least one ε4 allele e.g., ε2/4, ε3/4, or ε4/4) or allele negative (ε2/2, ε2/3, ε3/3).

### 2.3. Stimuli and Procedures

Odor threshold was measured utilizing N-butyl alcohol because it is a potent stimulus for the olfactory nerve, which does not impact the trigeminal nerve until it reaches high concentrations. Odorant n-butyl alcohol was prepared in a series of 10 dilutions, beginning with 4% deionized water, with each successive dilution being one-third the previous concentration. On a given trial, participants were presented with the odor and a blank stimulus. Subjects were asked to sniff each one and choose which one smelled stronger. An incorrect choice prompted an increased concentration to be used in the next trial. Thresholds were determined after four sequential correct choices. Thresholds were assessed in each nostril [45].

Odor identification was assessed with the San Diego Odor Identification Test (Murphy et al., 2002). The test utilizes 8 common household odors in opaque jars, and a picture board to aid in identification. Odors were presented at 45 s intervals to minimize adaptation. Participants were asked to close their eyes during the presentation of the stimulus to avoid visual cues. A visual illustration for each odorant (8) and for the distractor odors (12) was presented on the picture board, and participants were asked to determine which of the 20 pictures identified an odorant. A score was then calculated based on the number of correct responses ranging from 0 to 8 and converted to a percentile. The San Diego Odor Identification Test has been shown to have high test-retest reliability [63,64].

Odor familiarity was assessed utilizing a visual analogue scale with participants being asked to rate how familiar a presented odor was to them (e.g., soap and cloves), from not at all familiar to very familiar [35]. Stimuli were randomly chosen from a battery of 80 common odors presented for smelling with the eyes closed. These were not the same set as the set of odor items in the identification test.

The Mini-Mental State Examination (MMSE), widely-used in the screening of cognitive impairment in older adults, was administered. It includes assessments of attention, orientation, memory language and visual spatial skills [65]. Though previous research suggests variability in MMSE scores in AD patients [66], and other measures of cognitive integrity may be more sensitive and specific to cognitive changes [67], we chose to use it here for comparison because it is the most widely used clinical screening instrument.

### 2.4. Data Analysis

A ROC curve plots the trade-off between sensitivity and specificity. Area under the ROC curve (AUC) was used to determine diagnostic accuracy. An AUC ranges from 0 to 1; the higher the AUC, the better the prediction of conversion to the diagnostic category. Higher AUC values indicate better sensitivity, the ability to determine a that patient falls into a diagnostic category, and specificity, the ability to determine that a person does not fall into a diagnostic category. ROC analysis was conducted in SPSS 23, which generated values for sensitivity and specificity, as well as AUC.

## 3. Results

Table 1 presents the descriptive statistics for the nonconverters: cognitively normal age-matched controls, MCI and AD patients, ApoE ε4 non-carriers and ApoE ε4 carriers for age, odor familiarity, odor identification, odor threshold, MMSE and years of education.

The following describes the ability of olfactory measures and the MMSE to predict conversions from cognitively normal control to MCI, cognitively normal control to AD and MCI to AD in ε4 carriers and non-carriers. AUC values for tasks that were statistically significant predictors of conversion at the important points in the development of AD indicated in the tables in bold. The higher the value for the AUC, the better the prediction of conversion to the diagnostic category. Overall, there was a higher rate of conversion in ε4 carriers than non-carriers, F(1, 495) = 19.07, *p* < 0.05.

### 3.1. ROC Analysis Comparing Cognitively Normal Controls to Converters from Control to MCI

We investigated the ability of odor measures to distinguish healthy normal controls (NC) from those who were controls and went on to develop MCI. For the complete list of results, see Table 2.

#### 3.1.1. MMSE

The MMSE was not highly sensitive and specific for distinguishing normal controls from those who converted to MCI in the overall sample (AUC = 0.46, *p* > 0.05). In Apoe ε4 carriers (*n* = 25), the MMSE was even less readily able to distinguish controls from controls who converted to MCI (AUC = 0.39, *p* > 0.05). The AUC for both categories remained indeterminate.

#### 3.1.2. Odor Measures

Each of the odor measures had numerically higher AUC values for distinguishing those who remained healthy controls and those who converted to MCI than the MMSE, however the AUC values did not reach statistical significance. The AUC for each odor measure was as follows: familiarity 0.57, identification (ID) 0.51, ID and Familiarity together 0.57, threshold 0.54.

For ApoE ε4 carriers AUC for most odor measures did not reach statistical significance; the AUC was once again numerically higher than the MMSE for the following measures: ID 0.73, familiarity 0.70, familiarity and ID 0.80.

### 3.2. ROC Comparing Cognitively Normal Controls to Converters from Control to AD

We then reviewed the ability of odor measures to distinguish cognitively normal controls from those who went on to convert from cognitively normal control to AD. For results, please refer to Table 3.

#### 3.2.1. MMSE

The MMSE was not sensitive or specific in distinguishing cognitively normal controls from those who converted from control to AD in the overall sample. The AUC was 0.46 (*p* > 0.05). In Apoe ε4 carriers (*n* = 29), the ability of the MMSE to detect control to AD converters showed an AUC of 0.48 (*p* > 0.05). Once again, the MMSE was found to be indeterminate.

#### 3.2.2. Odor Measures

**Figure 2:**
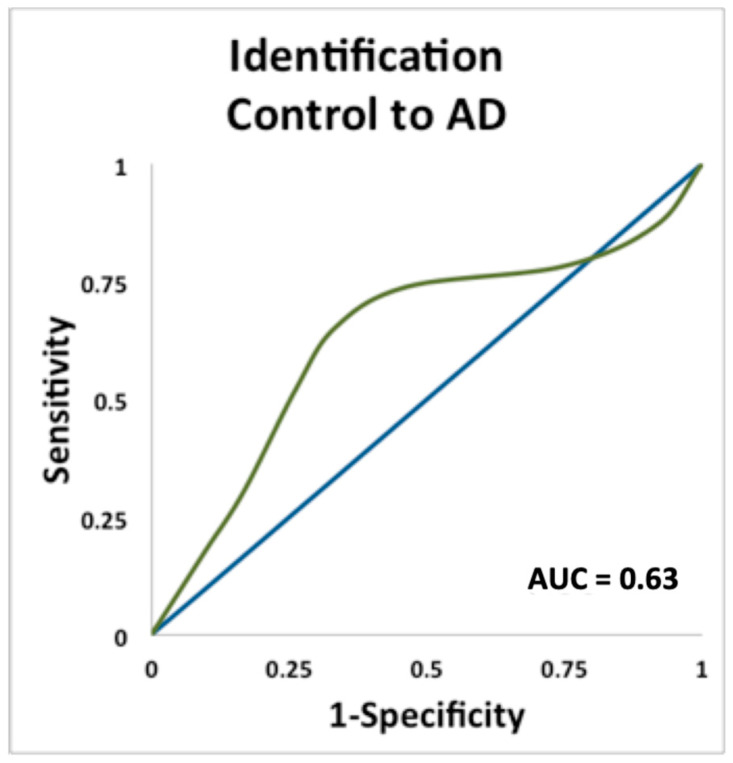
ROC curve plotting sensitivity and specificity for predicting conversion from cognitively normal controls to AD, using odor identification.

Odor ID was a significant predictor of conversion from cognitively normal control to AD (AUC = 0.63, *p* = 0.05) (See Figure 2). However, odor ID did not increase prediction in Apo ε4 carriers as it had in other conversion categories discussed above.

**Figure 3:**
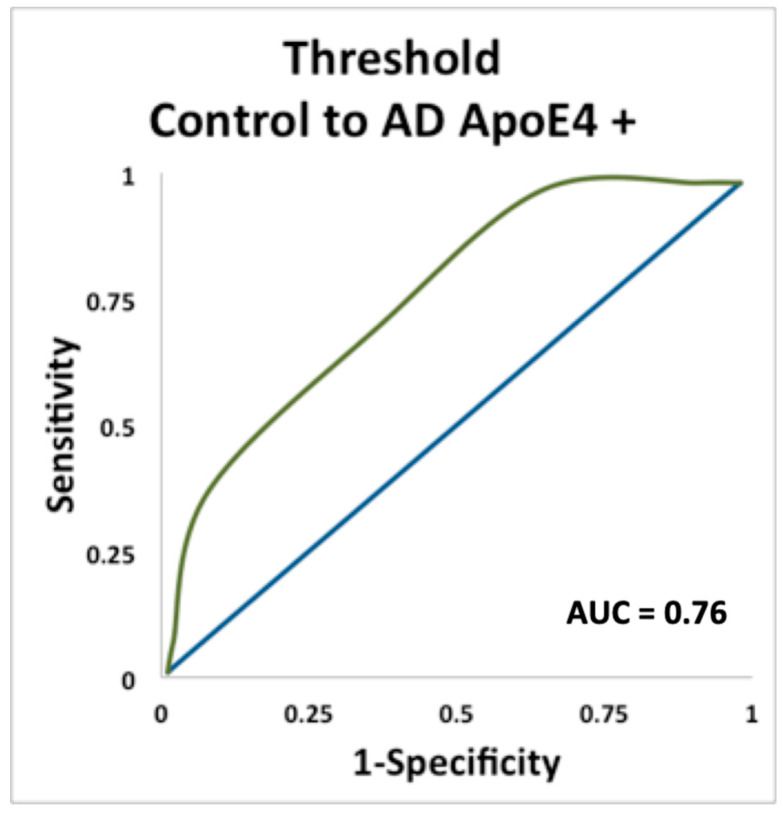
ROC curve plotting sensitivity and specificity for predicting conversion from cognitively normal controls to AD in carriers of the ε4 allele, using odor threshold.

For Apoe ε4 carriers, threshold was the only significant predictor of conversion from cognitively normal control to AD (AUC = 0.76, *p* = 0.029) (Figure 3).

### 3.3. ROC Analysis Comparing MCI and Converters from MCI to AD

We then examined the ability of odor measures to distinguish MCI (MCI) from those who were MCI and went on to develop AD (MCI to AD). For a complete list of results, see Table 4.

#### 3.3.1. MMSE

The MMSE was neither highly sensitive nor specific for distinguishing non-converter MCI patients from those who converted from MCI to AD in the overall sample (AUC = 0.53, *p* > 0.05). In Apoe ε4 carriers (*n* = 23), AUC = 0.58, *p* > 0.05. In both samples, the AUC remained indeterminate.

#### 3.3.2. Odor Measures

Odor familiarity, odor ID and familiarity, and threshold had higher AUC values for distinguishing those who remained in MCI and those who converted from MCI to AD. However, the results did not reach significance in the overall sample. The AUC for each odor measure was as follows: familiarity 0.61, ID and familiarity together 0.70, and threshold 0.54.

**Figure 4:**
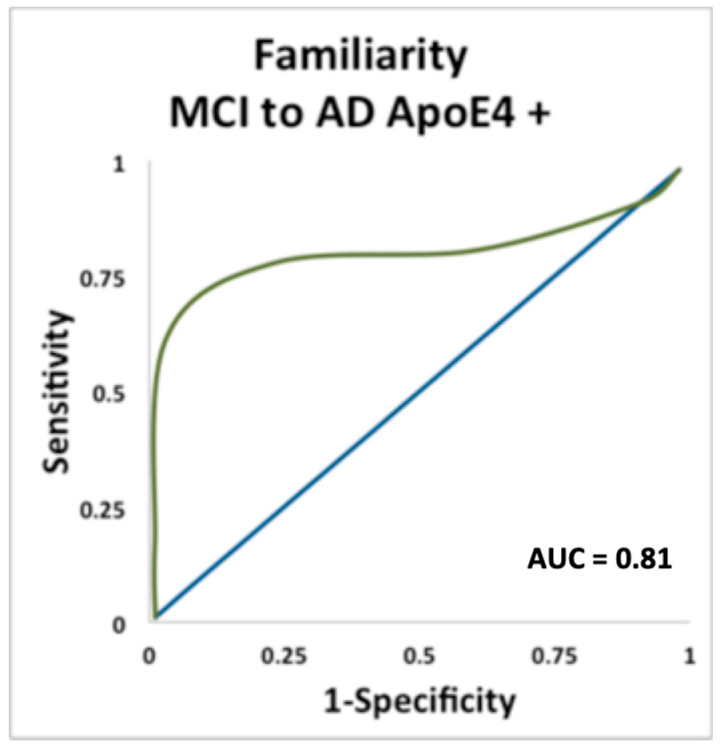
ROC curve plotting sensitivity and specificity for predicting conversion from MCI to AD in carriers of the ε4 allele, using the odor familiarity measure of remote odor memory.

**Figure 5:**
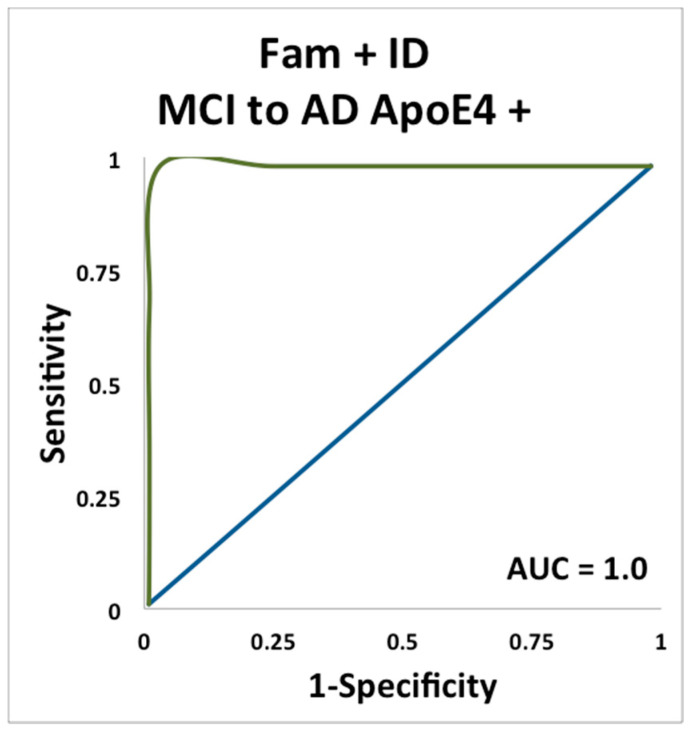
ROC curve plotting sensitivity and specificity for predicting conversion from MCI to AD in carriers of the ε4 allele, using both odor identification and the odor familiarity measure of remote odor memory.

For Apoe ε4+ carriers, both the familiarity (AUC = 0.81, *p* = 0.013) and familiarity and ID (AUC = 1.0, *p* = 0.034) measures reached asymptotic significance and were the strongest predictors of conversion from MCI to AD (Figure 4 and Figure 5). The AUC value for odor ID (AUC = 0.67) was numerically higher than the AUC for the MMSE, however it did not reach asymptotic significance.

### 3.4. Logistic Regression Analyses

ROC analyses were followed up by logistic regression on each of the measures: odor identification and familiarity, familiarity, identification, threshold and MMSE. Analyses controlled for gender, years of education and age. Table 5 presents these findings. Statistical significance is indicated in bold. Significant effects of the ROC analyses were confirmed and, additionally, familiarity was demonstrated to predict progression from control to MCI in ApoE 4+, identification predicted conversion from aMCI to AD in ApoE4+ and identification and familiarity were found to be a significant predictor of aMCI to AD overall. Note that for the combination of identification and familiarity for predicting conversion from aMCI to AD in the ApoE 4+ participants, there were no false positives and no false negatives, thus for the Wald statistic, 0 is recorded.

## 4. Discussion

The current results support the efficacy of using odor threshold, odor identification and odor familiarity tasks in predicting subsequent conversion from cognitively normal control to mild cognitive impairment and AD. These effects were particularly pronounced in Apoe ε4+ carriers. AUC values for tests that were statistically significant predictors of conversion at these important points in the development of AD are indicated in the tables for the ROC analyses in bold. Statistical significance is also indicated in bold in Table 5, which presents the results of the logistic regression analyses. A number of values that were numerically substantial were not statistically significant. We speculate that the numbers of converters in the analyses was a limitation. It is possible that in a larger sample additional tasks would produce statistically significant findings. The present results shed light on the olfactory tests that are significant predictors of conversion between states at important points in the development of AD.

Different olfactory measures were more sensitive and specific at different states. Establishing when odor testing might predict conversion between states may provide useful information for patient–practitioner clinical decision-making and pharmaceutical interventions.

### 4.1. Threshold

The current study demonstrated that odor threshold was able to distinguish cognitively normal controls from controls who converted to AD, in Apoe ε4 carriers (AUC = 0.76). Threshold was more readily able to distinguish these two groups than the MMSE which was indeterminate (AUC = 0.48). This is consistent with previous findings that odor threshold is associated with the degree of dementia in AD [34,45] and that Apoe ε4 carriers show more significant odor threshold deficits than non-carriers, particularly in the year before they convert to AD [29]. Poorer odor threshold in those who are converters might be expected to reflect underlying structural changes occurring in Apoe ε4+ carriers prior to the onset of clinical symptoms.

Overall, our results support the efficacy of an odor threshold task in predicting the conversion from cognitively normal control to Alzheimer’s disease in ε4 carriers. Finally, the AUC for threshold was found to be more sensitive and specific than the AUC for the MMSE in the ε4 carriers.

### 4.2. Identification

A large number of studies have reported differences in odor identification between cognitively normal controls and AD patients [37,39,47,68], as well as between cognitively normal controls and MCI or individuals who convert to a diagnosis of MCI (see [1] for a review). The current study found that odor identification alone produced a significant AUC of 0.63 for prediction of conversion from cognitively normal control to AD in the overall sample. Given the existing literature and the fact that successful odor identification is dependent on odor detection, odor memory, verbal semantic memory and knowledge of an odor, all processes that rely heavily on structures that show early AD pathology (e.g., entorhinal cortex), we had expected that it would be a significant predictor of conversion to AD.

Deficits in odor identification in families of AD patients might suggest genetic components in odor identification [50,51]. The present study demonstrates that odor identification impairment combined with familiarity was most predictive of conversion from MCI to AD in the presence of the ApoE ε4 allele. This supports previous research, which has found that deficits in odor identification occur earlier in Apoe ε4+ carriers [52]. It is of potential clinical relevance that combining odor identification with odor familiarity produced particularly high prediction of conversion from MCI to AD in Apoe ε4 carriers AUC = 1.0). Overall, the results suggest that Apoe ε4+ status combined with odor identification and familiarity scores might be used as an effective marker for predicting MCI to AD converters in trials of disease modifying drugs.

### 4.3. Odor Familiarity

AD patients rate odors as significantly less familiar than do cognitively normal controls, reflecting a deficit in odor familiarity [35]. The results from the current study support the hypothesis that odor familiarity is a sensitive and specific marker for ε4 carriers with MCI to convert to AD. Odor familiarity (AUC = 0.81) and the combination of odor familiarity plus odor identification (AUC = 1.0), but not odor identification or MMSE alone, were significant predictors of conversion from MCI to AD in the Apoe ε4 carriers, suggesting the potential for the combination of odor identification and familiarity to serve as a biomarker.

Familiarity is distinctly associated with structures within the medial temporal lobe that are the site of early AD pathology. The literature suggests that familiarity measured with non-olfactory tasks, is compromised in AD, in MCI and in Apoe ε4 carriers while being largely left intact in normal aging, though not every study is in agreement [69,70,71,72]. Resistance to normal aging coupled with compromise in AD and MCI suggests that familiarity is sensitive to developing AD. Odor familiarity may be particularly sensitive to prodromal AD because it relies heavily on these mesial temporal areas, which are critical for both familiarity and olfactory processing. The parahippocampal cortex is associated with recollection and retrieval of contextual information, whereas the perirhinal cortex contributes to and is necessary for familiarity-based tasks [73]. Human fMRI studies have revealed greater activation to familiar than to unfamiliar odors in the parahippocampus, middle and inferior temporal gyri and the parietal cortex incorporating the precuneus [74,75,76]. Both the mesial temporal areas and the precuneus are affected in MCI [41,77]. Activation within the right medial orbitofrontal area in NC has also been observed during tasks of odor familiarity. Rated familiarity during an fMRI task correlates with activation in the inferior frontal gyrus and the parahippocampal gyrus [74,78]. Many of the areas in the mesial temporal lobe and hippocampus are critical for successful retrieval of the memory trace or experiencing of familiarity. Hence, diminished performance on tasks of odor familiarity may reflect atrophy of the areas in the mesial temporal lobe that are affected in AD (entorhinal cortex, hippocampus). Additional research is needed to elucidate which areas are affected at different stages of disease progression and how these areas are associated with olfactory function.

Since familiarity of odors, more so than familiarity of faces, has been found to reflect a deficit in familiarity in people with AD [35], odor familiarity deficits might indicate a further progression of neuropathology within the hippocampal complex. This could be expected to be reflected in deficits in people who convert from cognitively normal to MCI and AD.

Odor identification and familiarity tests are rapidly administered, use common odor stimuli, and can be administered by non-experts, advantages that will render these tests useful in large population studies, clinical trials and diagnosis. As indicated above, combining odor identification with odor familiarity produced particularly high prediction of conversion from MCI to AD in Apoe ε4+ carriers, suggesting its potential utility in clinical practice and pharmaceutical trials.

### 4.4. Apoe ε4 Carriers

Apoe ε4 carriers consistently showed a greater likelihood of converting from cognitively normal control to MCI and AD. The MMSE was not as effective as odor measures in predicting Apoe ε4+ CN to MCI and MCI to AD converters from non-converters. ERP and fMRI studies suggest that Apoe ε4 carriers expend significantly more cognitive effort to compensate for olfactory deficiencies and show hyperactivation and structural differences in odor memory regions during successful performance on olfactory memory tasks [42,44]. Over time, hyperactivation is neurotoxic and would be expected to increase neuropathology in the areas of the olfactory network that overlap areas important in AD, further eroding both cognitive and olfactory performance. The current results support the hypothesis that Apoe ε4 carriers with odor impairment are more likely to convert from cognitively normal control to MCI or from MCI to AD. In cognitively normal ε4 non-carriers, the best predictor of future AD was odor identification. However, in carriers the best predictor was odor threshold. We might speculate that carriers of the ε4 allele have sustained more preclinical AD-related neuropathology in olfactory areas than non-carriers, and thus, the olfactory threshold may be impaired earlier.

### 4.5. MMSE

The study suggests that measures of olfactory memory may be more sensitive and specific for conversion to MCI and AD, particularly in ε4+ carriers, than the MMSE. This is consistent with some previous findings which suggest variation between patients with regard to their MMSE scores [65] and that other measures of cognitive integrity might be more sensitive and specific to detecting changes [67]. Although the MMSE is used to help diagnose dementia and for screening in primary care practice, studies have shown that its sensitivity and specificity depend on a number of factors, including demographics [79]. Previous work has shown that in assessment at the ADRC, the MMSE had more noise than the DRS, showed a moderate floor effect and a slight ceiling [80]. It was only one of a battery of tests of cognitive function used for diagnosis in the ADRC. We had used it here because of its widespread use in screening. While the MMSE has clinical utility in screening for AD, combined odor memory measures might be more effective measures of early detection and tracking the progression of the disease.

### 4.6. Limitations

The present results shed light on the olfactory tasks that are the best predictors of conversion at important points in the development of AD. A number of AUC values that were numerically substantial were not statistically significant. We speculate that the numbers of converters in the analyses presented a limitation. It is possible that in a larger sample additional tasks would produce statistically significant findings. Future studies would benefit from larger sample sizes and an ethnically diverse participant pool. In addition to limitations, the current study also had a number of notable strengths, including a well-characterized sample of individuals from the UCSD ADRC who were diagnosed as cognitively normal, having mild cognitive impairment or Alzheimer’s disease, and who were assessed with a number of objective olfactory tests using published methods.

### 4.7. Conclusions

The current study investigated the sensitivity and specificity of measures of odor threshold, odor familiarity and identification in predicting transitions between cognitive states; in particular for Apoe ε4+ individuals. The results reveal novel information on the olfactory tasks that are the best predictors of conversion at these important transition points in the development of AD. Because olfactory processing relies on the mesial temporal areas that show the earliest neuropathological changes in prodromal AD, olfactory perception reflects underlying pathology during the earliest stages of AD. Different olfactory tasks can be expected to involve different neural networks for successful performance. Hence, a combination of two olfactory measures might be expected to produce higher sensitivity and specificity. In the current study, the combination of odor identification and odor familiarity produced an AUC of 1.00. Future research with psychophysical and neuroimaging techniques is needed to address important questions regarding the underlying neural networks that support these olfactory tasks and the progression of degeneration in AD. Additional research comparing the sensitivity and specificity of these olfactory tasks to CSF biomarkers and structural imaging biomarkers will also be of great interest.

Odor familiarity, threshold and identification each present the potential for a non-invasive low-cost screening tool that can be administered by non-experts. Combining odor identification with odor familiarity produced particularly high prediction of conversion from MCI to AD in Apoe ε4 carriers. These measures are easily and quickly administered, suggesting utility for practitioners in preliminary screening for risk of cognitive impairment prior to a comprehensive assessment for diagnosing MCI or AD, or in enriching samples in clinical trials with individuals likely to convert from MCI to AD. Since many individuals are not candidates for MRI or lumbar puncture or do not have access to scanning technology, odor testing may fulfill a significant medical need.

## Figures and Tables

**Table 1:** Descriptive Statistics.

AD Status	n	Age	Familiarity	Identification	Threshold	MMSE	Years of Education
		Mean (SE)	Mean (SE)	Mean (SE)	Mean (SE)	Mean (SE)	Mean (SE)
Cognitively Normal	170	72.97 (0.80)	98.05 (2.65)	5.01 (0.23)	5.76 (0.17)	24.01 (0.92)	15.17 (0.29)
Mild Cognitive Impairment	42	73.89 (1.25)	74.59 (5.02)	3.35 (0.52)	5.05 (0.38)	23.37 (1.22)	15.00 (0.42)
Alzheimer’s Disease	210	74.32 (0.57)	72.75 (2.22)	2.86 (0.17)	4.64 (0.16)	20.80 (0.44)	14.73 (0.42)
ApoE4 Non-Carrier	124	72.79 (0.81)	92.97 (3.09)	4.21 (0.30)	5.60 (0.21)	24.68 (0.71)	15.30 (0.29)
ApoE4 Carrier	132	71.66 (0.73)	83.82 (3.08)	3.81 (0.21)	5.30 (0.21)	22.40 (0.69)	14.25 (0.27)

**Table 2:** AUC values for odor measures and MMSE: Controls converting to MCI.

	Control to MCI	Control to MCI in Apoe ε 4+
ID + Familiarity	0.57	0.80
Familiarity	0.57	0.70
ID	0.51	0.73
Threshold	0.54	0.59
MMSE	0.64	0.39

**Table 3:** AUC values for odor measures and MMSE: Cognitively normal controls converting to AD.

	Control to AD	Control to AD in Apoe ε 4+
ID + Familiarity	0.56	0.52
Familiarity	0.52	0.57
ID	0.63 *	0.59
Threshold	0.5	0.76 *
MMSE	0.46	0.48

* Values in bold are statistically significant. Note. The higher the AUC, the better the prediction of conversion to the diagnostic category.

**Table 4:** AUC values for odor measures and MMSE: MCI converting to AD.

	MCI to AD	MCI to AD in Apoe ε 4+
ID + Familiarity	0.7	1.00 *
Familiarity	0.61	0.81 *
ID	0.52	0.67
Threshold	0.54	0.38
MMSE	0.53	0.58

* Values in bold are statistically significant. Note. The higher the AUC, the better the prediction of conversion to the diagnostic category.

**Table 5:** Logistic regression: odor measures and MMSE.

	**Control to MCI**			**Control to MCI in ApoE4+**		
	**Wald**	**SE**	**Sig**	**Wald**	**SE**	**Sig**
**ID + Familiarity**	0.407	0.01	0.524	2.719	0.069	0.099
**Familiarity**	1.276	0.008	0.259	4.499	0.054	**0.034**
**ID**	0.058	0.184	0.809	1.812	0.871	0.178
**Threshold**	0.452	0.11	0.502	2.743	0.237	0.097
**MMSE**	0.008	0.026	0.93	0.415	0.045	0.519
	**Control to AD**			**Control to AD in ApoE4 +**		
	**Wald**	**SE**	**Sig**	**Wald**	**SE**	**Sig**
**ID + Familiarity**	0.628	0.017	0.428	0.702	0.029	0.402
**Familiarity**	3.076	0.005	0.079	2.496	0.02	0.114
**ID**	7.973	0.017	**0.005**	1.039	0.329	0.308
**Threshold**	0.834	0.097	0.361	5.397	0.457	**0.02**
**MMSE**	0.313	0.023	0.576	1.045	0.052	0.307
	**aMCI to AD**			**aMCI to AD in ApoE4 +**		
	**Wald**	**SE**	**Sig**	**Wald**	**SE**	**Sig**
**ID + Familiarity**	5.755	0.032	**0.016**	0	0	**<0.01**
**Familiarity**	2.811	0.008	0.094	5.207	0.019	**0.022**
**ID**	1.057	0.231	0.304	5.943	0.134	**0.015**
**Threshold**	0.917	0.135	0.338	1.35	0.243	0.245
**MMSE**	0.763	0.037	0.382	1.051	0.07	0.388

Values in bold are statistically significant.

## Data Availability

The data presented in this study are available upon request from the corresponding author.
